# COVID-19: Social Distancing and Physical Activity in United Kingdom Residents With Visual Impairments

**DOI:** 10.1177/0145482X221108703

**Published:** 2022-11

**Authors:** Clare Strongman, Patrick Swain, Henry Chung, Viviane Merzbach, Dan Gordon

**Affiliations:** 1 Cambridge Centre for Sport and Exercise Sciences, 150697 Anglia Ruskin University, Cambridge, UK; 2 Faculty of Health and Life Sciences, 5995 Northumbria University, UK

**Keywords:** COVID-19, visual impairment, blindness, physical activity, public health

## Abstract

**Introduction:** The introduction of the COVID-19 lockdown and social distancing policy has the potential to restrict access to physical activity, change exercise behavior, and to increase sedentary behavior. This study was conducted with the support of British Blind Sport and evaluates the effect of the lockdown policy on adults with visual impairments in the United Kingdom (UK). **Methods:** An online survey based on the World Health Organization’s Global Physical Activity Questionnaire was completed by 73 participants (median age 35–44 years, 59% female) to gain information on how the implementation of the lockdown policy by the UK government has affected physical activity and sedentary behavior in adults that are visually impaired. Paired sample t-tests and Wilcoxon signed rank tests were used to analyze continuous and Likert scale data, respectively. **Results:** The majority of participants continued to exercise during lockdown, but the nature of this activity altered with a significant change to exercising in a private indoor space (+190% (always), *z* = −3.871, *p* < 0.001), and those exercising alone (+118% (always), *z* = −2.595, *p* = 0.009). The volume of activity reduced in all cases, between −11% and −52%, with significant changes in duration of vigorous day-to-day activity, moderate day-to-day activity, travel by foot or cycle, and vigorous recreational activity. Sedentary behavior increased on average by 21% (*t*(59) = −2.050, *p* = 0.045), with a greater effect seen in females (+36%, *t*(35) = −2.525, *p* = 0.016). **Discussion:** Reductions in physical activity volume and increases in sedentary behavior are consequences of the UK lockdown for those with visual impairments. The health and well-being implications of these data to this specific population are of particular concern. **Implications for Practitioners:** Lockdown measures should be designed with consideration of the needs of people with disabilities. Innovative ways to engage these populations in physical activity are strongly encouraged.

Currently, there are ∼350,000 people in the United Kingdom (UK) who are registered as visually impaired: acuity less than 6/60 (<20/200) with a full field of vision, acuity of up to 6/24 (20/80) with a moderate reduction of field, and acuity of 6/18 (20/60) or a large part of the field of vision is missing (RNIB, 2019). Of this group, 173,735 are classified as severely impaired with an acuity of less than 3/60 (<10/200) with full visual field, or less than 6/60 (<20/200) with severe reduction of field (e.g., tunnel vision), or greater than 6/60 (>20/200) with very reduced field of vision (RNIB, 2019). Individuals with a visual impairment have been shown to exhibit lower outcomes for quality of life compared to age-matched people without vision loss, in areas such as access to physical activities, limitations in activities of daily living, depression, anxiety, and other health-related outcomes (Evans et al., 2007; Holmstrom, 2020; Horowitz et al., 2005; Kempen et al., 2012). Holbrook et al. (2009) demonstrated that, on average, adults with visual impairments are completing less physical activity compared to the general population. This reduced level of activity is important as Haegele et al. (2017) demonstrated that, within adults with visual impairment, physical activity was a significant predictor of health-related quality of life. Additionally, Łabudzki and Tasiemski (2013) established that physical activity was significantly correlated with life satisfaction in individuals that are visually impaired.

With the outbreak and consequent spread of the COVID-19 virus, the world is experiencing a global pandemic, which has led to major changes in typical day-to-day living (WHO, 2020). In the UK, as in many countries, a policy of social distancing accompanied by a national lockdown of society was introduced on March 23, 2020, in an attempt to control the spread of the virus (Brown, 2020). As part of this prevention mechanism, the public were instructed to minimize contact with others and undertake no more than one session of exercise in a public space per day. Aside from this daily exercise period, the other forms of activity permitted were for essential reasons only: the collection of medications and food. Although the rationale for this approach is clear in its attempt to reduce and control the spread of COVID-19, little is known about the effect of these profound disruptions to daily life, on both mental and physical well-being.

Social distancing procedures that have been implemented in public spaces are predominately visual, and this focus on visual communication may dramatically affect individuals who are visually impaired and potentially contribute to reduced independence and increased social isolation (RNIB, 2020). In addition, the government policy of only allowing close contact with members of one’s own household could further affect this population, making access to a human guide outside of one’s immediate household impossible, and further limiting access to outdoor spaces and physical activity. Charities such as Sport England and British Blind Sport have promoted online content to fill this gap in provision (British Blind Sport, 2020); despite suggestions, online exercise may be unsuitable for people with visual impairments (BBC, 2020). Although the lockdown policy has affected the whole UK population, the measures implemented may not be appropriate for people with visual impairments and reduce accessibility and inclusion for this group.

Public Health England (2020) highlights the importance of staying physically active during lockdown as a means of maintaining health and well-being. However, little is known about how people with visual impairments will respond to the limitations imposed on social mobility and the consequent effects on both physical activity and sedentary behavior in this already at-risk group. It is hypothesized that social distancing measures will contribute to reduced activity and increased sedentary behavior in people with visual impairments. Therefore, the purpose of this study was to quantify the physical activity and sedentary behavior in individuals with visual impairment before and during the first UK COVID-19 lockdown.

## Methods

### Participant Recruitment

Participants were included in the study if they were 18 years old or over, a UK resident social distancing due to COVID-19, had no change in their health status since the start of the lockdown on March 23, 2020, and had one or more visual impairments. *Visual impairment* was classified according to the RNIB guidance (RNIB, n.d.), with visual acuity and visual field descriptions for each classification listed within the survey questions.

Participants gave their informed consent to participate via a questionnaire that was available online at OnlineSurveys.ac.uk. The online questionnaire was based on the WHO Global Physical Activity Questionnaire (GPAQ) version 2 (GPAQ, n.d.), which gathers information on frequency and duration of day-to-day physical activity, recreational activity, and sedentary behavior. Questions asked participants to enter the frequency of activity (for example, “How many days per week did you exercise to online content?” with the options of selecting 0–7 days per week) and the number of hours for each in free text boxes as well as gathering information on the type of sports via multiple choice. In addition, Likert scale items were used to evaluate the different types of recreational behavior, such as how often the participant exercised alone. The survey was adapted by the addition of questions on exercise modality and behaviors, and the nature of visual impairment. The GPAQ survey has been demonstrated to have fair-to-moderate validity (Chu et al., 2015) and its use allowed a wide reach when lockdown measures restricted other means of obtaining information.

The survey was open between April 27 and May 31, 2020, and ethical approval was granted by Anglia Ruskin University research committee. Participants were asked to evaluate the nature and duration of their participation in physical activity and exercise prior to the lockdown period compared to their current (during lockdown) activity. Although this short delay introduces the possibility of recall bias affecting the pre-lockdown results, this effect was minimized due to the survey being carried out shortly after the lockdown was imposed, limiting incorrect recall and maximizing accuracy of results.

Participants were recruited via social media and via email messages that were sent to members of charities focusing on increasing participation in individuals with visual impairment. To increase accessibility, the online survey was designed for optimal access by people with visual impairments (Papadopoulos & Goudiras, 2005), including the use of large print and enhanced color contrast. The questionnaire was also tested using common screen readers to check accessibility, and completion of the survey via telephone was also offered to increase participation and reduce selection bias.

### Statistics

Alpha was set to 0.05 for all tests, and all analyses were done in SPSS (version 26; IBM SPSS Statistics). Unless stated, significance, mean, and standard deviations are reported in each case. Likert data were analyzed via Wilcoxon signed rank test. Paired sample t-tests were used to compare data prior and during lockdown, following the central limit theorem (Kwak & Kim, 2017) suggesting that parametric tests are valid for a large sample size.

## Results

### Participants

Of those surveyed, 59% were female, and 53% were married. The median age was 35–44 years (27%), and 79% reported having a severe visual impairment. The majority of participants surveyed did not take part in competitive sport, but 28 identified as being amateur athletes (competing in sports events for no pay) and two as professional (paid) athletes. Participants reported a range of conditions contributing to their visual impairment with the most common being nystagmus (12%), retinitis pigmentosa (12%), and cataracts (9%); other conditions (12%) included tumors, Stargardt’s disease, and injury.

Information on ethnicity was not collected due to the lack of racial diversity in the UK, with 2011 census data reporting 86% of people living in England and Wales identified as belonging to the White ethnic group (Office for National Statistics, 2012), making subgroup analysis inappropriate due to insufficient sample sizes for other ethnicities.

### Participation in Physical Activity

Overall, participation in exercise remained unchanged with 81% of participants stating that they took part in “fitness, exercise, or sport” prior to lockdown with 97% of those continuing to do so after lockdown. The type and duration of this exercise changed, but the majority remained able to participate in some form of physical activity during lockdown.

Prior to the lockdown policy, 8% of participants reported taking part in online exercise, which increased to 38% following the introduction of restrictions. However, only 64% of those who took part in online exercise following the lockdown considered the content to be friendly to visually impaired people.

Physical activity data before and during lockdown are presented in Table 1, with any significant results marked.

**Table 1.** Physical Activity Levels Before and During Lockdown.

Parameter	Before lockdown	During lockdown	*n*	*t*
Vigorous activities during day-to-day living				
Frequency (days per week)	1.21 (2.05)	0.99 (0.27)	68	1.193
Duration (hours per day)	0.57 (1.22)	0.30 (0.93)	71	3.205**
Volume (hours per week)	2.23 (4.73)	1.42 (3.91)	71	2.455*
Moderate activities during day-to-day living				
Frequency (days per week)	3.38 (2.82)	2.34 (2.87)	68	3.364**
Duration (hours per day)	1.04 (1.61)	0.62 (1.16)	69	2.001*
Volume (hours per week)	5.52 (9.74)	3.18 (5.88)	69	1.961
Travel by foot/cycle				
Frequency (days per week)	4.31 (2.33)	2.68 (2.82)	65	4.464**
Duration (hours per day)	1.04 (0.94)	0.54 (0.69)	63	3.876**
Volume (hours per week)	5.50 (5.40)	2.63 (4.12)	63	4.079**
Vigorous intensity recreational activity				
Frequency (days per week)	1.81 (1.89)	1.73 (2.35)	67	0.317
Duration (hours per day)	0.90 (1.50)	0.50 (0.93)	67	2.825**
Volume (hours per week)	2.68 (6.34)	2.10 (4.30)	67	1.137
Moderate activity recreational activity				
Frequency (days per week)	1.91 (2.15)	2.08 (2.56)	66	−0.498
Duration (hours per day)	0.92 (1.68)	0.65 (1.31)	64	1.809
Volume (hours per week)	3.61 (9.65)	3.20 (8.48)	64	0.589

*Note*: *p < 0.05; **p < 0.01.

### Day-to-Day Physical Activity

#### General

Both moderate and vigorous activities during daily living decreased during lockdown, both in terms of frequency and duration. The frequency of vigorous activity decreased nonsignificantly (−18%) and the duration was reduced on average by approximately 20 minutes. Moderate physical activity within day-to-day living reduced significantly with a 31% decrease in frequency (one day less per week) and a 40% decrease in duration (approximately 40 minutes less per day).

#### Biological Sex

Subgroup analysis (see Appendix A) showed that participation in vigorous day-to-day activity was not significantly affected by biological sex: *t*(29) = 1.676, *p* = 0.104 males; *t*(40) = 1.771, *p* = 0.084 females. However, females had significantly decreased moderate day-to-day activity, −66%, *t*(38) = 2.771, *p* = 0.009, whereas males increased their moderate physical activity on average (+42%) but this effect was not statistically significant, *t*(29) = −0.998, *p* = 0.327.

#### Marital Status

Participation in vigorous day-to-day activity was more affected in married participants, with significant effects in both duration and volume, −54%, *t*(37) = 2.864, *p* = 0.007; −49%, *t*(37) = 2.589, *p* = 0.014, respectively, whereas single people were not significantly affected overall with an increase in the frequency of participation, +12%, *t*(27) = −0.619, *p* = 0.541, but a decrease in duration, −29%, *t*(30) = 1.985, *p* = 0.056.

#### Athletic Status

Participants who took part in competitive sporting events had a statistically significant decline in the duration of vigorous day-to-day activity: −38%, *t*(29) = 2.447, *p* = 0.021. Participants who did not compete as athletes had a significant decrease in duration of both vigorous, −59%, *t*(40) = 2.148, *p* = 0.038; and moderate activity, −46%, *t*(39) = 2.039, *p* = 0.048.

#### Travel Via Foot or Cycle

##### General

Travel via foot or cycle decreased significantly in both frequency and duration, with a loss of 1.6 days per week of activity—38%, *t*(64) = 4.464, *p* < 0.001—of approximately 30 minutes per day: −48%, *t*(62) = 3.876, *p* < 0.001.

##### Biological Sex

Travel changes were significant for females, with a decrease in both frequency, −48%, *t*(36) = 4.802, *p* < 0.001; and duration -57%, *t*(35) = 4.012, *p* < 0.001. There were no significant effects for males.

##### Marital Status

Significant effects were observed in both frequency and duration of travel for both married, −42% days/week, *t*(34) = 3.129, *p* = 0.004; −53% hours/day, *t*(35) = 2.650, *p* = 0.012; and single people, −35% days/week, *t*(27) = 3.079, *p* = 0.005; −42% hours/day, *t*(25) = 3.581, *p* = 0.001.

##### Athletic Status

There were no significant changes in travel frequency or duration when comparing people who competed in sporting events and those who did not compete.

#### Recreational Activity

##### General

Overall, vigorous recreational activity decreased in frequency and duration, with a significant reduction of approximately 24 minutes per day, −44%, *t*(66) = 2.825, *p* = 0.006, but moderate recreational activity increased slightly in frequency, +9%, *t*(65) = −0.498, *p* = 0.620, but reduced in duration, −29%, *t*(63) = 1.809, *p* = 0.075.

##### Biological Sex

The change in duration was significant for females in particular, with an average reduction of 25 minutes per day of vigorous intensity recreational activity: *t*(39) = 2.685, *p* = 0.011. Overall, females participated in 10% more hours per week spent engaging in moderate-intensity recreational activity, but this was not statistically significant. Although males experienced a decrease in recreational activity, this finding only showed a significant effect in duration of moderate physical activity with a reduction on average of 37 minutes per day—*t*(26) = 2.195, *p* = 0.037—with no other significant differences found.

##### Marital Status

Single people showed a significant overall decrease in the duration of vigorous physical activity, −55%, *t*(27) = 2.889, *p* = 0.008, with a reduction of 24 minutes per day. Single people also participated more often in moderate recreational activity over lockdown (+10% days per week) but this finding was not statistically significant. There were no other significant differences found in participation in recreational activity in single and married people

##### Athletic Status

In athletes, the decrease in duration of recreational physical activity was significant for both vigorous and moderate activity: −55%, *t*(25) = 2.982, *p* = 0.006; −47%, *t*(24) = 2.405, *p* = 0.024, respectively. In those participants who did not compete in sporting events, there were no significant effects seen in changes in duration or frequency of recreational activity.

#### Sedentary Behavior

Sedentary behavior is shown in Table 2, with subgroup analysis by biological sex, marital status, and athletic status (professional and amateur athletes have been combined due to sample size). Any significant effects are marked.

**Table 2.** Sedentary Behavior Levels Before and During Lockdown.

Group		Before lockdown (hrs)	During lockdown (hrs)	*n*	*t*
All		6.19 (4.51)	7.46 (5.24)	60	−2.050*
Biological sex	Male	6.53 (4.20)	6.43 (5.40)	21	0.214
	Female	5.97 (4.76)	8.14 (5.12)	36	−2.525*
Marital status	Single	7.13 (4.89)	8.24 (5.54)	25	−1.134
	Married	5.48 (4.22)	6.89 (5.11)	34	−1.710
Athletic	Athlete	5.88 (4.51)	6.66 (5.09)	25	−0.997
Status	Non-athlete	6.41 (4.57)	8.02 (5.36)	35	−1.784

*Note*: *p < 0.05.

Sedentary behavior significantly increased on average by 21%, *t*(59) = −2.050, *p* = 0.045, translating to an approximate increase of 80 minutes per day. Subgroup analyses revealed that sedentary behavior was particularly pronounced in females with an average increase of 36%, or ∼135 minutes per day, *t*(35) = −2.525, *p* = 0.016, whereas males reported an overall nonsignificant reduction, −2%, *t*(23) = 0.124, *p* = 0.903. There were no other significant subgroup differences.

#### Exercise Modality

Before lockdown, the most popular exercise modalities were fitness cardio machines (36%), walking (34%), and bodyweight training (29%). During lockdown, the most popular were walking (52%), weight training (32%), and running (21%).

#### Days and Times

In general, the trend toward participating in physical activity “any day of the week” increased during the lockdown by 73%, as opposed to fixed days of the week. There was also a corresponding decline in participation on specific days during lockdown.

Similarly, there was a trend showing increased exercise at “any time of day,” with decreased evening activity (−40.0% between 5 p.m. and 9 p.m.) and a corresponding increase in late afternoon (+53.3% between 3 p.m. and 5 p.m.). Exercising at a fixed time of day slightly decreased during lockdown.

#### Recreational Behavior

Participants were asked to evaluate their activity in specific venue types, and relating to exercise structure, to assess the change in behavior and how this change related to lockdown. This information is shown in Table 3.

**Table 3.** Likert Scale Data for Recreational Behavior Before and During Lockdown.

	Never (1)	Seldom (2)	Sometimes (3)	Frequently (4)	Always (5)	Median (IQR)	*z*
Private indoor environment (*n* = 44)							
Before (%)	34.6	11.5	13.5	26.9	13.5	3 (1–4)	−3.871**
During (%)	7.8	5.9	5.9	41.2	39.2	4 (4–5)	
Private outdoor environment (*n* = 40)							
Before (%)	54.0	12.0	22.0	10.0	2.0	1 (1–3)	−1.215
During (%)	42.2	13.3	22.2	22.2	0.0	2 (1–3)	
Outdoors in public (*n* = 44)							
Before (%)	28.0	4.0	20.0	32.0	16.0	3 (1–4)	−0.502
During (%)	30.2	1.9	17.0	32.1	18.9	4 (1–4)	
Follow an exercise schedule (*n* = 53)							
Before (%)	34.0	7.5	13.2	28.3	17.0	3 (1–4)	−1.297
During (%)	47.9	6.3	10.4	16.7	18.8	2 (1–4)	
Use fitness apps/trackers (*n* = 44)							
Before (%)	40.4	19.2	3.8	13.5	23.1	2 (1–4)	−0.136
During (%)	52.1	8.3	2.1	8.3	29.2	1 (1–5)	
Exercise alone (*n* = 47)							
Before (%)	22.2	9.3	20.4	31.5	16.7	3 (2–4)	−2.595*
During (%)	10.9	1.8	27.3	23.6	36.4	4 (3–5)	

*Note*: *p < 0.05; **p < 0.01

Prior to closure, 66% of the participants reported frequently or always attending leisure centers, with 11% frequently or always attending outdoor activity centers and multiuse games areas. This change also was reflected in participants’ exercise behavior with an increase of 190% always exercising in private during lockdown, and an increase of 53% frequently exercising in private, this change in behavior was statistically significant (*z* = −3.871, *p* < 0.001). A similar effect can also be seen in the reported numbers of participants always exercising alone during lockdown, which showed a change of 118%, with a significant effect (*z* = −2.595, *p* = 0.009).

## Discussion

The UK lockdown has imposed restrictions limiting time spent outside the home, nonessential travel, and allowing only one outing for exercise per day. The lockdown has affected the whole population, but, when combined with existing barriers to exercise, visual social distancing measures, and lack of access to public transport, this effect may have had a greater impact on people with visual impairments. Reducing physical activity and increasing sedentary time may have adverse health and well-being implications.

Although measures have been taken to reduce selection bias and remove confounding factors, it is recognized that these data captured a snapshot of UK adults with visual impairments during a period of extreme conditions, so may have limited generalizability outside of this scope. Therefore, these data highlight important, but preliminary, trends that can inform discussions and practices for policy-makers and health practitioners.

### Day-to-Day Physical Activity

Both moderate and vigorous activities during daily living decreased as a result of the lockdown, both in terms of frequency and duration. Subgroup analysis (see Appendix A) showed that participation in vigorous day-to-day activity was not significantly affected by biological sex; however, females had significantly decreased moderate day-to-day activity (−66%), whereas males increased their moderate physical activity on average (+42%), but this change was not statistically significant. This discrepancy between biological sexes may relate to increased childcare responsibilities due to school closures, which work is still predominantly done by women (Ascher, 2020). In addition, Hughes (2020) reported increased house and garden maintenance over lockdown and suggested these activities may have replaced recreational activities, which may partly account for these changes.

### Travel Via Foot or Cycle

Significant changes in travel via foot or cycle were seen in both frequency and duration, with a loss of 1.6 days per week of activity (38%) of approximately 30 minutes per day (−48%). This finding was expected, due to the travel restrictions imposed by the lockdown, only allowing essential travel. This measure is also likely to affect people with visual impairment more than the general population, since use of public transportation is more common within this group, providing “the only feasible mobility option” (Sáez et al., 2019, “Abstract”, para. 1) to access both work and recreational activity.

### Recreational Activity

Despite a reduction in recreational activity during lockdown, people with visual impairments report exceeding the recommended 150 minutes of moderate physical activity per week (WHO, 2004). However, self-reported duration of activity may be unreliable (Schaller et al., 2016); participants tend to overestimate their participation; thus, this value may be misleading. Comparison of before and during lockdown, therefore, reduces self-reporting bias and gives a relative picture of the effects of lockdown, to which participation in recreational activity reduced. The implications of these findings indicate from available evidence that there would have been an associated decrease in health-related benefits (WHO, 2004) and a decrease in health-related quality of life (Haegele et al., 2017).

The majority of people who exercised prior to lockdown continued to do so once restrictions were put in place, but there was a trend toward movement from group activity toward individual participation. The closure of gyms and leisure centers during the lockdown (Legislation.gov.uk, 2020) limited the exercise opportunities or facilities available to people with visual impairment. Participants’ exercise behavior shows a significant increase in people exercising in private and exercising alone during lockdown. This behavior change may reflect government advice that restricted access to outdoor exercise to once per day, the lack of availability of a human guide, or difficulties in complying with social distancing measures. In addition, the shift to indoor private spaces may be due to the increase in use of online content. Overall, vigorous recreational activity decreased, but moderate recreational activity increased slightly, a change that may relate to government guidelines to increase moderate recreational activity, but limiting exercise to one session per day until restrictions were relaxed on May 13, 2020, to allow unlimited outdoor exercise (Brown, 2020).

The trend toward participating in physical activity “any day” increased during the lockdown by 72%, as opposed to fixed days of the week, indicating a more flexible approach. This trend may be a result of changes in working conditions (e.g., furlough) and lack of structured exercise routines (e.g., gym classes). There was also a similar trend showing increased exercise at “any time,” with decreased evening activity (−40% between 5 p.m. and 9 p.m.) and a corresponding increase in late afternoon (+53% between 3 p.m. and 5 p.m.).

Jaarsma et al. (2014) found key barriers to participation in recreational physical activity were lack of transport and dependence on others. The lockdown policy also limited access to facilities, human guides, and caregivers. Limiting nonessential travel and contact with members from other households may have significantly increased these barriers for people with visual impairments to access recreational activity.

### Sedentary Behavior

Sedentary behavior significantly increased by approximately 80 minutes per day. Subgroup analyses revealed that increased sedentary behavior was particularly pronounced in females. The reason for these differences by sex are likely multifaceted; however, the health implications of these data are of concern, since sedentary time of greater than 7 hours a day is associated with increased all-cause mortality risk (Ku et al., 2018). Further, there is evidence to indicate that participants underestimate sedentary time when self-reporting (Kastelic & Šarabon, 2019), suggesting that these data may be more pronounced if measured objectively (e.g., accelerometer), and, therefore, may underestimate the actual health risk.

## Conclusion

Decreased participation in physical activity was evident in a visually impaired population, particularly affecting females, with decreases in day-to-day activity, travel, and vigorous intensity recreational activity. There was an increase in the frequency of moderate recreational activity, with a concurrent reduction in session duration. Activities such as exercising with others were replaced by exercising alone and in a private indoor space. Time spent participating in sedentary behavior significantly increased in females by approximately 135 minutes a day, yet remained largely unchanged in males.

### Implications for People With Visual Impairments

People with visual impairments should consider the arrangement of support to encourage continued physical activity participation to maintain physical and mental health and to reduce social isolation. In addition, people with visual impairments can provide valuable feedback on online resources and accessibility of available physical activity options to minimize negative effects of future restrictions.

### Implications for Practitioners

As the UK government relaxes lockdown measures (UK Government, 2020), it is important that policy-makers be made aware of these data and their implications and begin to speculate what measures can be taken to ensure specific populations, such as those with visual impairments, are not disproportionately affected by current or new lockdown restrictions or both. Health practitioners should continue to encourage and support participation in physical activity throughout the week and develop accessible ways to engage people with visual impairments in activity. These measures need to remain accessible, even under heavy lockdown restrictions, such as closure of sport and leisure centers. Practitioners should attempt to engage women and single people, in particular, since these subgroups were most seriously affected by the lockdown measures.
